# A mechanically robust and moisture-retentive hydrocolloid dressing for chronic wound management

**DOI:** 10.1038/s41598-026-64229-w

**Published:** 2026-08-12

**Authors:** Laila Waled, Nour. H. S. Habib, Alaa Farid, Roaa Ibrahim, Noha Elnagar, Soha M. Kandil, Gehan Safwat, Mohamed Taha

**Affiliations:** 1 Faculty of Biotechnology, October University for Modern Sciences and Arts (MSA), Giza, 12566 Egypt; 2 Nano Gate Company, 9254 Hoda shaarawy, Al Abageyah, El Mukkatam, Cairo, 11571 Egypt; 3 Electronic and Magnetic Materials Department, Advanced Materials Division, Central Metallurgical Research and Development Institute (CMRDI), Cairo, 11421 Egypt; 4 Department of Pharmaceutical Biotechnology, Faculty of Biotechnology, Badr University, Assiut, Egypt; 5 Department of Pharmaceutics and Drug Manufacturing, Faculty of Pharmacy, Modern University for Technology & Information (MTI), Cairo, 12055 Egypt

**Keywords:** Hydrocolloid, Wound dressing, Silver NPs, Anti-inflammatory, Biotechnology, Materials science, Microbiology, Nanoscience and technology

## Abstract

Due to their prolonged healing times and susceptibility to infection, chronic wounds pose a significant clinical challenge. In order to improve the treatment of chronic wounds, a silver-curcumin (Ag-Cur) hydrocolloid dressing was developed in this work. integrates nanotechnology with polymer-based wound dressings by using Ag-Curcumin nanoparticles (Ag-Cur NPs) as a nano filler, which were prepared and characterized using various techniques to confirm successful formation of stable silver nanoparticles with the acceptable properties, including surface Plasmon resonance (SPR), zeta potential (ZP), dynamic light scattering (DLS), Poly dispersive index (PDI), and HR-TEM. The results demonstrated the formation of Ag-Cur nanoparticles with a hydrodynamic size of 197.3 ± 0.709 nm and a zeta potential of -27.5 ± 0.603 mV. Morphological analyses via SEM and EDX revealed a porous structure with uniform nanoparticle distribution, while mechanical testing demonstrated superior tensile strength and a swelling ratio of 428.76%, surpassing commercial dressings. The hydrocolloid dressings shown high batch-to-batch reproducibility, and the Ag–Cur nanoparticles demonstrated acceptable long-term stability. Biological evaluations showed significant antimicrobial activity against pathogens such as *Staphylococcus aureus*, *Escherichia coli*, and *Candida albicans*, alongside potent anti-inflammatory effects (82.43% albumin denaturation inhibition at 1000 µg/mL) and substantial antibacterial, bactericidal, and anti-biofilm activity against MRSA, highlighting its promise as a multifunctional wound dressing for infection management and reducing biofilm-related healing delay. The sustained release profile, reaching 80% after 100 h, ensures prolonged therapeutic effects. Finally, with a CC_50_ of 470.06 ± 23.09 µg/mL, the Ag-Cur hydrocolloid demonstrated moderate cytocompatibility with HSF-1 cells. Additionally, Ag-Cur-HCD succeeds over both Blank-HCD and Ag-HCD in suppressing LPS-induced inflammation and oxidative stress in HSF-1 cells, as seen by the *IL-1β*,* TNF-α*, and NO levels. As a result, Ag-Cur-HCD considerably modulates important inflammatory and antioxidant pathways. These results suggest that the Ag-Cur hydrocolloid shows potential for future wound dressing applications. Future studies will focus on evaluating the formulation in models of infected and chronic wounds, as well as long-term safety and comparative efficacy assessments.

## Introduction

Chronic wounds affect the populations globally, causing significant clinical and economic challenges to the current healthcare system; around 2% of the populations suffers with chronic wounds, which are becoming prevalent due to an aging population, high obesity, and diabetes^1^. In addition to impairing patients’ quality of life, managing chronic wounds causes an enormous financial load on healthcare systems; in 2022, $148.65 billion was spent on wound care^2^. Chronic wounds include venous leg ulcers, diabetic foot ulcers, and pressure ulcers^3,4^. These wounds usually persist for months or even years and are distinguished by a failure to follow the typical phases of healing (haemostasis, inflammation, proliferation, and remodelling) within the expected period^1^. Persistent bacterial infections, excessive inflammatory reactions, poor angiogenesis, and underlying comorbidities like diabetes, vascular disease, or immobility are all factors that contribute to their chronicity^3,5^. The main functions of conventional wound care techniques, including gauze dressings, adhesive bandages, or simple absorbent pads, are to protect the wound and absorb exudate. However, the complicated pathophysiological challenges to healing in chronic wounds, such as microbial colonization, biofilm development, and persistent inflammation, are frequently overlooked by these traditional dressings^1,3,6^. To enhance the clinical healing, novel wound care products that combine antibacterial, anti-inflammatory, and regenerative properties must be presented. Recent developments in nanotechnology have revolutionized the management of wounds by allowing it feasible to develop bioactive dressings that contain nanoparticles exhibiting particular therapeutic properties^7^. Silver nanoparticles (AgNPs) have attracted significant attention due to their broad-spectrum antimicrobial activity against pathogens commonly associated with chronic wound infections and multidrug-resistant bacteria, such as *Pseudomonas aeruginosa*^8^, *Staphylococcus aureus*^9^, *Escherichia coli*^10,11^, and *methicillin-resistant Staphylococcus aureus* (MRSA)^12^. Silver ions disrupt bacterial cell membranes, interfere with DNA replication, and inhibit metabolic processes, making silver-based dressings a staple in managing infected wounds. However, excessive silver use can lead to cytotoxicity and delayed healing, highlighting the need for controlled delivery systems that maximize antimicrobial benefits while minimizing toxicity^13,14^. Silver nanoparticles (AgNPs) with high antimicrobial efficacy, attributed to their confinement effect and controlled silver ion release, were widely used for different biomedical applications. However, the cytotoxicity is combined with AgNPs, highlighting the need for further research into green nanotechnology and alternative topical delivery systems such as films and hydrogels^15–17^. Furthermore, curcumin, the natural polyphenol extracted from the turmeric plant (Curcuma longa), has attracted attention due to biological features, which include antioxidant, anti-inflammatory, and acceleration of wound-healing^18–21^. However, the low bioavailability, poor water solubility, and rapid degradation under physiological conditions of curcumin limited its clinical application. Hence, nanotechnology can be used to resolve tese challenges by conjugating them into nano-delivery systems^22^. Previous studies have illustrated the potential of silver-curcumin nanoparticles to exhibit high antimicrobial and anti-inflammatory properties compared to their native components^23^. Recently, AgNPs and curcumin were investigated in various wound dressing scaffolds, including hydrogels^24–26^, and nano-fibres^27–30^. For example, Gupta et al. (2020) fabricated bacterial cellulose hydrogels matrices embedded with curcumin-cyclodextrin-stabilized silver nanoparticles, demonstrating antimicrobial activity and biocompatibility^26^. However, hydrogels usually require a second dressing layer, have little mechanical strength, and some types need to be moistened often to maintain their useful characteristics^31,32^. While electrospun nanofibers have a high surface area and controlled drug release, their production is complex and less practical for widespread clinical use compared to the solvent casting method employed for hydrocolloids^33,34^. Du et al., 2025 investigate on curcumin-loaded silver-based metal–organic frameworks for burn wounds^35^, Despite having strong antibacterial and antioxidant effects for the healing of infected wounds, their practical clinical uses may be limited by issues including complicated synthesis, potential cytotoxicity, low mechanical strength, and expensive production costs^36,37^. Hydrocolloids have practical advantages over nanofibrous scaffolds^27–30^ or conventional hydrogels^24–26^, such as ease of application, patient comfort, and compatibility with normal wound care protocols.

The aim of this study is to integrate silver-curcumin (Ag-Cur) nanoparticles into a hydrocolloid matrix to represent a novel approach that integrates nanotechnology with polymer-based wound dressings to harness the synergistic benefits of both components. Firstly, the green formula of silver-curcumin nanoparticles will be prepared and assessed for its physicochemical characterizations, including Zeta potential, DLS, PDI, optical absorption, and TEM. Then, incorporated into a hydrocolloid patch to investigate its physicochemical properties, including morphology, mechanical features, swelling behaviour, and in vitro release study, as well as its biological activity, including antimicrobial effect against Gram-positive bacteria, Gram-negative bacteria, fungi, MRSA with MIC, MBC, and anti-biofilm activity; anti-inflammatory activity; as well as biocompatibility with human skin fibroblasts (HSF-1).

## Experimental work

### Materials

Carboxymethyl cellulose (CMC), sodium alginate (SA), calcium chloride dihydrate (CaCl_2_·2H_2_O), Polyethylene glycol 400 (PEG 400), Phosphate-buffered saline (PBS, pH 7.4), Tween 80, glycerol (purity ≥ 99.5%), Curcumin (purity ≥ 98%, derived from Curcuma longa), and xanthan gum were supplied from LOBA-CHEMIE PVT.LTD, India. Silver nitrate (AgNO_3_, purity ≥ 99.0%) was obtained from Merck, Germany. Microbial strains including *Candida albicans (ATCC 10231) Staphylococcus aureus (ATCC 25923)*,* Staphylococcus epidermidis (ATCC 12228)*,* Escherichia coli (ATCC 25922)*, and *Pseudomonas aeruginosa (ATCC 27853)* were obtained from the American Type Culture Collection (ATCC). Human skin fibroblast cells (HSF-1, CRL-1502) were sourced from ATCC for cytotoxicity testing. Culture media, including nutrient agar and Mueller-Hinton agar, were supplied from Thermo-Fisher Scientific, USA. Fatal bovine serum (FBS) and MTT (3-(4,5-dimethylthiazol-2-yl)-2,5-diphenyltetrazolium bromide) assay kits were also obtained from Thermo-Fisher Scientific, USA, for cell culture and viability assessments. Dimethyl sulfoxide (DMSO) and trypan blue dye were purchased from Sigma (St. Louis, Mo., USA). Fetal bovine serum, DMEM, HEPES buffer solution, L-glutamine, gentamycin and 0.25% Trypsin-EDTA were purchased from Lonza (Belgium).

All materials were used without additional purification. Double-distilled water (DDH_2_O) was utilized to prepare the solutions.

### Preparation methods

#### Preparation of silver-curcumin nanoparticles

The synthesis of silver-curcumin (Ag-Cur) nanoparticles was adapted from a green chemistry approach to ensure environmental sustainability and biocompatibility, as described by Abdellah et al.^23^. Curcumin (0.1 g) was dissolved in 10 mL of PEG 400 at 60 °C under constant stirring at 500 rpm for 30 min using a magnetic stirrer (Thermo Scientific™ Cimarec+™ Hotplate, USA). Silver nitrate (0.0016 g) was then added to the Cur-PEG solution, and the mixture was stirred at 60 °C for an additional 2 h. The reaction was monitored visually for a color change from yellow to brownish-yellow, indicating the formation of Ag-Cur nanoparticles due to the reduction of silver ions by curcumin’s phenolic groups^23^. The resulting suspension was left to cool at room temperature.

#### Preparation of Ag-Cur hydrocolloid dressing

The solvent casting approach was used for the fabrication of hydrocolloid patches. Briefly, 0.75 g of SA was dissolved in DDH_2_O (100 mL) at 40 °C with stirring for 1 h to form a clear solution. Then, 0.037 g of CaCl_2_·2H_2_O was dissolved in 2 mL of DDH_2_O and added dropwise to the previous solution with stirring (800 rpm) for 30 min, forming a stable gel matrix after. Glycerol was then added as a plasticizer to enhance the flexibility of the hydrocolloid then; 0.75 g of CMC was dissolved in 100 mL of DDH_2_O at 50 °C with stirring at 500 rpm for 45 min to ensure complete solubility. Xanthan gum (0.7 gm) was gradually added to the CMC solution under continuous stirring to avoid clumping, and the mixture was stirred for an additional 1 h to achieve homogeneity. The SA and CMC-xanthan gum solutions were mixed and stirred at 400 rpm for 30 min to form a homogeneous solution. Ag-Cur nanoparticles were incorporated into these portions at concentrations of 7.5% (w/v). The mixture was then poured into sterile Petri dishes (150 mm diameter) and dried in a hot air oven (DAIHAN^®^ Forced-air Drying Oven, South Korea) at 65 °C for 12 h to form flexible hydrocolloid patches. A control hydrocolloid (blank) without Ag-Cur nanoparticles was prepared similarly for comparison. The dried patches were cut into 2 cm × 2 cm and stored in sterile, airtight containers at 4 °C until further analysis.

### Characterization techniques

#### Physico-chemical characterizations

To verify the optical absorption of curcumin and surface Plasmon resonance (SPR) of Ag-Cur nanoparticles, a UV-Vis spectrophotometer (Cary series UV-Vis-NIR, Australia) was used. Spectra were recorded from 200 to 600 nm with a resolution of 1 nm. Particle size distribution, the hydrodynamic diameter, was determined using DLS, and zeta potential of Ag-Cur nanoparticles was assessed using a Malvern Zetasizer Nano ZS (UK). Nanoparticle suspensions (0.1 mg/mL in DDH2O) were sonicated for 5 min to prevent aggregation and analyzed at 25 °C.

The morphology and size of Ag-Cur nanoparticles were examined using a JEM-2100 TEM (JEOL -Japan). A drop of diluted nanoparticle suspension (10 ppm) was placed on a carbon-coated copper grid, air-dried, and imaged at an accelerating voltage of 200 kV.

FTIR/ATR spectra were collected on Vertex 70-RAM II (Bruker Analytical, Germany) at room temperature, covering a 4000 –400 cm^− 1^ range. The crystalline structure and phase identification were investigated via Empyrean Malverpanalytical, Netherland X- ray diffraction, at (Kα) = 1.54060 Å with 2-Theta (5.0° − 80°), with step size 0.04°. Furthermore, the surface morphology and elemental composition of the hydrocolloid patches were analyzed using an FEI Quanta 250 SEM equipped with an EDAX detector (USA).

Additionally, the tensile strength and ductility of the hydrocolloid films were performed using a Zwick tensile testing machine Z010 (Load Cell 1 kN), Germany. Film strips (5 cm × 1 cm) were clamped and stretched at a crosshead speed of 5 mm/min until failure. The Young’s modulus (E-modulus) and elongation at break were calculated from stress-strain curves, with measurements conducted in quintuplicate to ensure statistical reliability.

#### Swelling capacity

The fluid absorption capacity of the hydrocolloid was determined by immersing pre-weighed film samples (2 cm × 2 cm) in PBS (pH 7.4) at 37 °C. Samples were removed at intervals (1, 2, 4, 6, …and 50 h), blotted dry, and weighed^38^. The Swelling Capacity was calculated using the formula:

1 $${\text{Swelling~\% }}={\mathrm{~}}\frac{{{{\mathrm{W}}_{\mathrm{t}}} - {{\mathrm{W}}_{\mathrm{i}}}}}{{{{\mathrm{W}}_{\mathrm{i}}}}} \times 100$$

where (${{\mathrm{W}}_{\mathrm{t}}}$) is the weight of the swollen patch and (${{\mathrm{W}}_{\mathrm{i}}}$) is the dry weight. Experiments were performed in triplicate.

#### In vitro release study

Firstly, a calibration curve for Ag-Cur NPs was measured, ranging from 1 to 20 µg/mL then, evaluate Ag-Cur NPs release as follow: 1.0 g of hydrocolloid patch was dispersed in 100 mL of phosphate-buffered saline (pH 7.4) containing 1% (v/v) Tween 80. The mixture was incubated at 37 °C. Tween 80 is used to create sink conditions and guarantee that the hydrophobic active ingredients are properly solubilized during the release time and prevent drug precipitation and maintain a concentration gradient enough for accurate release profiling. The UV-Vis absorbance of the released Ag-Cur NPs was measured at 425 nm to determine the released concentration of Ag-Cur NPs at time t (C). The cumulative release percentage of Ag-Cur was calculated using the following equation:

2 $${\text{Cumulative release }}\% {\text{ }}=\frac{{{\mathrm{C~}}\left( {\mathrm{t}} \right)}}{{{\mathrm{C~}}\left( {\mathrm{i}} \right)}}*{\mathrm{1}}00$$

where:

${\mathrm{C~}}\left( {\mathrm{i}} \right)$ is the initial amount of Ag-Cur NPs loaded in the hydrocolloid patch (g). C (t) is the released amount of Ag-Cur NPs (g) at time t.

Furthermore, to elucidate the release mechanism of Ag–Cur from the HCD hydrocolloid membrane, the release data will be fitted to the most common kinetic models: Zero-order model, First-order model, Higuchi model, Hixson Crowell model, and Korsmeyer–Peppas model.

### Biological assays

#### Antimicrobial assay

The antimicrobial activity of as-prepared materials was conducted using the modified well diffusion technique against Gram-negative bacteria *(Escherichia coli and Pseudomonas aeruginosa)*, Gram-positive bacteria *(Staphylococcus aureus and Staphylococcus epidermidis)*, and *Candida albicans*. Positive controls were performed using ketoconazole as the standard antifungal agent and gentamicin as the standard antibacterial agent^39^.

#### Anti-inflammatory activity

The albumin denaturation technique was used to assess the anti-inflammatory potential of a compound by measuring its ability to inhibit or reduce the denaturation of albumin. An albumin denaturation assay was performed based on the procedure described by Williams et al.^40^, This assay was suggested as a simple, ethical, and cost-effective alternative to in vivo animal testing for the preliminary screening of potential anti-inflammatory agents. Briefly, a protein denaturation assay was carried out using bovine serum albumin (BSA) (CN: 232936-2); the reaction mixture was prepared as follows, different concentrations of Ag-Cur hydrocolloid patch (0.5……., 15.6, …, 125, ., 500, and 1000 µg/mL) were mixed with BSA (0.5 mL, 1.5 mg/mL) and incubated at 37 °C for 20 min. After that, reaction mixtures were heated for 3 min at 57 °C, and 250 µL of 0.5 M phosphate buffer (pH 6.3) was mixed to each reaction mixture. Once the molecules in each reaction mixture had been distributed evenly, 100 µL of each mixture was transferred into different test tubes. Next, 1% (v/v) Folin-Ciocalteu’s reagent and copper-alkaline reagent were added in the same volumetric proportion. After the tubes were incubated for 10 min at 55 °C, they were allowed to cool, and a microplate reader (Thermo-Fisher-Scientific, Waltham, MA, USA) was used to measure absorbance at 650 nm. Recorded measurements were evaluated using different concentrations of diclofenac sodium as a reference drug, which was tested under the same conditions for absorbance determination. The inhibition of protein denaturation percentage was calculated by using the following relationship:

3 $$\% {\text{ inhibition}}\,=\,{\mathrm{1}}00{\text{ x }}{{\mathrm{V}}_{\mathrm{t}}}/{\text{ }}{{\mathrm{V}}_{\mathrm{c}}}-{\text{ 1}}$$

Where V_t_ is the absorbance of the tested material and V_c_ is the absorbance of control. Using the dose-response curve, the active material concentration for 50% inhibition was calculated by graphing the percentage inhibition versus the treatment concentrate and the control.

#### In vitro antimicrobial activity against MRSA

Using a modified disc diffusion method, the tested compound’s antibacterial efficacy against resistant Gram-positive bacterial species (Methicillin-Resistant Staphylococcus aureus) was evaluated^41^. 10 mL of fresh media (Mueller-Hinton agar) was used to cultivate 100 µl of the test bacteria until they achieved a count of about 108 cells/mL. A 100 µl microbial sample was applied to agar plates that matched the broth in which they were kept, and the disc diffusion method was used to test for susceptibility. After that, the plates were incubated at 37 °C for 24 h. The microorganism’s growth was noted following incubation. The obtained inhibition zone diameters provided a criterion for the antibacterial activity and were measured in millimetres. If an organism is sensitive to the compound, it will not grow in the vicinity of the well if it is placed on the agar. “Zone of inhibition” or “Clear zone” refers to this region surrounding the disc where there is no growth. The inhibitory action of the compound being studied is directly correlated with the size of the clear zone. Every experiment included solvent controls (DMSO) as negative controls. The tested drug was dissolved in DMSO, which revealed no inhibition zones, indicating that it had no effect on the tested microorganisms’ ability to grow. Ciprofloxacin, a standard antibacterial drug, was also used for positive control. The Ag–Cur -HCD- was tested at 10 mg conc. Ciprofloxacin was tested at 1 mg conc. as a positive control.

#### Determining Minimal Inhibitory Concentration (MIC) and minimum bactericidal concentration (MBC)

The MIC is the lowest antimicrobial agent concentration at which the organism’s growth in the micro-dilution wells is totally inhibited^42^. An overnight-grown pure culture of MRSA diluted to a concentration of 1 × 10^5 to 1 × 10^6 cfu/mL in growth-supporting broth (tryptic soy broth). The antimicrobial Ag-Cur-HCD is a diluted stock solution that is approximately 100 times more concentrated than the anticipated minimum inhibitory concentration (MIC).

(I) In 96-well microtiter plates, double-folded dilutions (1000 − 1.95 ug/mL) are prepared using medium. Broth with brain-heart infusion.
(II) To prevent Ag-Cur-HCD turbidity, a negative control consists of repeated double-folded dilutions (1000 − 1.95 ug/mL) without test organism inoculation to create a baseline.
(III) A baseline concentration of the microorganism is determined by plating an aliquot of the positive control.
(IV) After that, the microtiter plates are incubated for 16 to 20 h at 35 ± 2 °C.
(V) As long as there is no sample compromise in the ability to discern growth in the wells, the turbidity was identified by the naked eye and measured OD at 630 nm using a BioTek 800 TS microplate reader to enable reading microdilution tests and recording data.
(VI) Finally, Compare the amount of growth in the wells containing the antimicrobial agent with the amount of growth in the growth-control wells (no antimicrobial agent) used in each set of tests when determining the growth end points. To determine the MBC, the dilution representing the MIC and at least two of the more concentrated test product dilutions are plated and enumerated to determine viable CFU/mL. The MBC is the lowest concentration that demonstrates a pre-determined reduction (such as 99.9%) in CFU/mL when compared to the MIC dilution.

#### Microtitre plate assay for biofilm quantification

In 96-well polystyrene flat-bottom plates, the sample’s effect on biofilm formation was assessed^43^. Briefly, each well of the microplate was filled with 300 μL of inoculated fresh trypticase soy yeast broth (TSY) (final concentration 106 CFU/mL) and cultured in the presence of previously determined sublethal concentrations (75, 50, and 25% of MBC). Controls included medium-containing wells, drug-free wells, and methanol-only wells. For 48 h, plates were incubated at 37 °C. Following incubation, the supernatant was removed, and each well was carefully cleaned with sterile distilled water to eliminate any free-floating cells. Plates were then allowed to air dry for 30 min, and the biofilm that had developed was stained for 15 min at room temperature using a 0.1% aqueous solution of crystal violet. After incubation, the plate was cleaned three times with sterile distilled water to eliminate any remaining discoloration. After 15 min of incubation, the dye attached to the cells was dissolved by adding 250 μL of 95% ethanol to each well. The absorbance was then measured using a microplate reader at a wavelength of 570 nm.

4 $${\mathrm{Biofilm~inhibition~ability~of~sample~}}=\frac{{(1 - {\mathrm{~}}\left( {{\mathrm{abs}}.{\mathrm{~~sample~}} - {\mathrm{~~abs}}.{\mathrm{~~Blank}}} \right)}}{{\left( {{\mathrm{abs}}.{\mathrm{~~control~}} - {\mathrm{~abs}}.{\mathrm{~~Blank}}} \right)}}~ \times 100$$

#### Cytotoxicity assay

HSF-1 cells (human foreskin fibroblast cell line) were obtained from the American Type Culture Collection (ATCC, Rockville, MD, USA). Cells were maintained at 37 °C in a humidified atmosphere with 5% CO_2_ and sub-cultured twice weekly. The test sample was placed in a sterile Falcon tube containing 1 mL of DMEM and incubated for 24 h before addition to the HSF-1 cell line. HSF-1 cells were suspended in DMEM at a concentration of 5 × 10^5^ cells/mL and seeded into Corning^®^ 6-well tissue culture plates, followed by incubation at 37 °C with 5% CO_2_ for 24 h. The test compounds were added to the 6-well plates (in triplicate) at the desired concentrations. Three vehicle controls containing only media were included for each 6-well plate. After 24 h of incubation, MTT assay was used to assess the cell viability^44^. The 6-well plates’ culture medium was taken out and replaced with fresh RPMI 1640 medium free of phenol red. Each well, including untreated controls, received a 12 mM MTT stock solution (5 mg MTT in 1 mL PBS). The plates were then incubated for four hours at 37 °C with 5% CO_2_. After removing the medium, 500 µL of DMSO was added to each well, carefully mixed, and incubated for 10 min at 37 °C. A Sunrise, Tecan, Männedorf, Switzerland, microplate reader was used to measure the optical density (OD) at 590 nm.

The percentage of cell viability was calculated using the formula:

5 $$\% {\text{ Viability }}={\text{ }}\left[ {\left( {{\text{ODt }}/{\text{ ODc}}} \right)} \right]{\text{ }} \times {\text{ 1}}00$$

where ODt is the mean optical density of wells treated with the test sample, and ODc is the mean optical density of untreated control wells. The 50% cytotoxic concentration (CC_50_) was determined from dose-response curves generated using GraphPad Prism software (San Diego, CA, USA)^45^.

#### Determination the Human Tumor necrosis factor α (*TNF-α*), Human Interleukin 1-beta (*IL-1β*), and NO levels

The purpose of the experiment was to determine whether the tested material may protect HSF-1 cells from damage caused by LPS. After two hours of incubation with the tested sample, HSF-1 cells were stimulated with 10 µg/mL of LPS for twenty-four hours^46,47^. As previously noted, the levels of *TNF-α*, NO, and IL-1beta were investigated to better understand the inflammatory responses.

Nitric Oxide Assay, ELISA Kit Cat No, SL1275Hu, (Elabscience, Houston, TX, USA), Human NF-κB ELISA kit (Catalog No. MBS450580), and ELISA Kit Cat No. SL0984Hu for *IL-1β* assay.

Data are presented as mean ± SD (n = 5 independent experiments).

### Statistical analysis

The obtained results were expressed as mean ± SD which were evaluated by One-Way Analysis of Variance (ANOVA) and followed by the Tukey test for multiple comparisons using Graph Pad Prism (version 8.0; Graph Pad Software Inc. San Diego, California, USA). For statistical significance, values of *p* < 0.05 were considered. (*p < 0.05, **p < 0.01, and ***p < 0.001).

## Results and discussion

### Physico-chemical characterizations

**Fig. 1:**
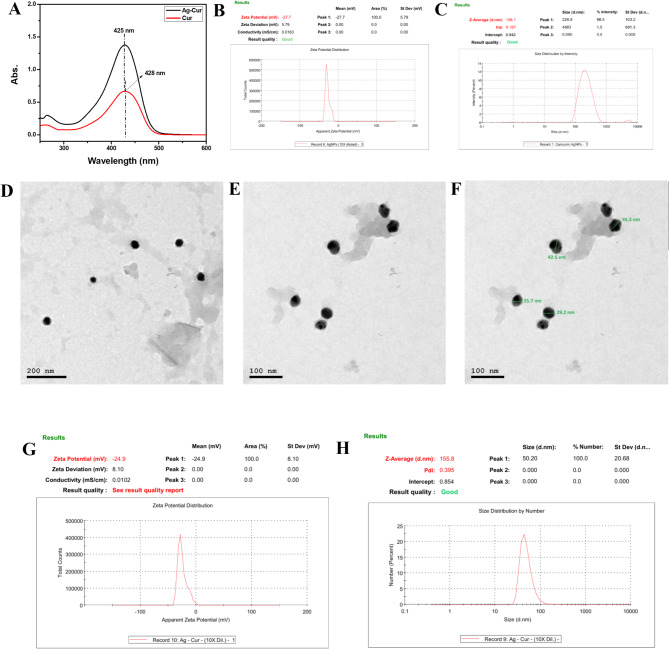
The physicochemical characterizations of Ag-Cur NPs (A) surface Plasmon resonance, (B) Zeta potential, (C) DLS, D, E, and F) TEM images inset figure selected area electron diffraction pattern. G) the zeta potential and H) the DLS of Ag-Cur NPs after 3-months of preparation. Respectively.

The synthesis of silver-curcumin (Ag-Cur) nanoparticles via a green approach, utilizing curcumin as a reducing agent for silver nitrate, was a strategic choice for this study. While the PEG 400 was used as a solvent to solubilize curcumin. PEG 400 also acts as a capping agent to enhance the nanoparticle stability by forming a protective layer around them, reducing aggregation and improving dispersion within the hydrocolloid matrix^48,49^. Figure 1A. Depicts the optical absorption of pure curcumin and Ag-Cur. Pure curcumin showed a broad band with a maximum absorbance peak at a wavelength of 428 nm, which could be assigned to low energy π–π* excitation of the curcumin, while the characteristic surface Plasmon resonance (SPR) peak of Ag-Cur nanoparticles was red-shifted to approximately 425 nm, suggesting the successful formation of Ag nanoparticles within curcumin^49,50^. The stability of nanoparticles and their likelihood of aggregation in the medium were evaluated by measuring the zeta potential (ZP) (Fig. 1B). The ZP value of the prepared curcumin-silver nanoparticles (Ag-Cur NPs) was − 27.5 ± 0.603 mV; these findings are closely related to previously reported values for curcumin-coated silver nanoparticles^51^. These results is indicate stable nanoparticles due to steric hindrance provided by polyethylene glycol (PEG), which was used as a capping agent during Ag-Cur NPs synthesis to prevent agglomeration, and suggest moderate colloidal stability, with the negative charge likely imparted by curcumin’s phenolic groups, which prevent aggregation through electrostatic repulsion^52^. Additionally, dynamic light scattering (DLS) analysis provided insights into the nanoparticle characteristics, confirming their particle size in the nanoscale range. The hydrodynamic diameter of 197.3 ± 0.709 nm (Fig. 1C) is very close to the previous study^26^. According to earlier reports, values between 0.1 and 0.4 are regarded as moderately polydisperse and homogenous, and the synthesized Ag-Cur s’ PDI value of 0.193 ± 0.013 shows moderate polydispersity in colloidal phase^26^. Furthermore, the morphology and particle size of Ag-Cur NPs were evaluated using TEM. Figure 1 (D-F) displayed TEM micrographs of Ag-Cur NPs with a spheroidal shape and an average diameter of 35 ± 10 nm. The polydispersity index (PDI) value of 0.193 ± 0.013, combined with TEM images, indicates a moderately polydisperse synthesis of Ag-Cur s, reflecting a moderate size distribution consistent with the TEM observations. The diameter of the nanoparticles resulting from the TEM investigation was expected to be smaller since TEM image analysis offers the hard sphere diameter while DLS presents the hydrodynamic diameter of a nanoparticles, it was shown that kinetic stability is provided by a size lower than 200 nm, hence preventing droplet aggregation and gravitational separation^6,53,54^. TEM images further confirmed the formation of homogeneous, spheroidal Ag-Cur NPs. Kinetic stability at sizes below 200 nm effectively prevents droplet aggregation and gravitational separation. Selected area electron diffraction (SAED) patterns of the Ag-Cur NPs confirmed their crystalline nature (inset figure, Fig. 1F)^55^.

Additionally, after three months of storage in ambient conditions, the physicochemical characteristics of the produced Ag–Cur nanoparticles were re-examined to further assess their colloidal stability. When compared to the freshly prepared formulation, the nanoparticles’ zeta potential of − 25.13 ± 1.66 mV Fig. 1G), hydrodynamic diameter of 164.23 ± 7.44 nm (Fig. 1H), and PDI of 0.295 showed very slight modifications. The Ag-Cur NPs stayed within the nanoscale range with a moderate size distribution, exhibiting acceptable colloidal stability, despite the minor change in the amplitude of the zeta potential and in the particle size, suggesting low particle aggregation during storage. The formulation components’ efficient steric stabilization, which reduces particle agglomeration and maintains dispersion homogeneity over time, is responsible for this stability. These findings indicate that the developed Ag–Cur NPs possess acceptable storage stability under ambient conditions, supporting their suitability for further incorporation into the hydrocolloid dressing and potential biomedical applications.

### Ag-Cur hydrocolloid dressing

**Fig. 2:**
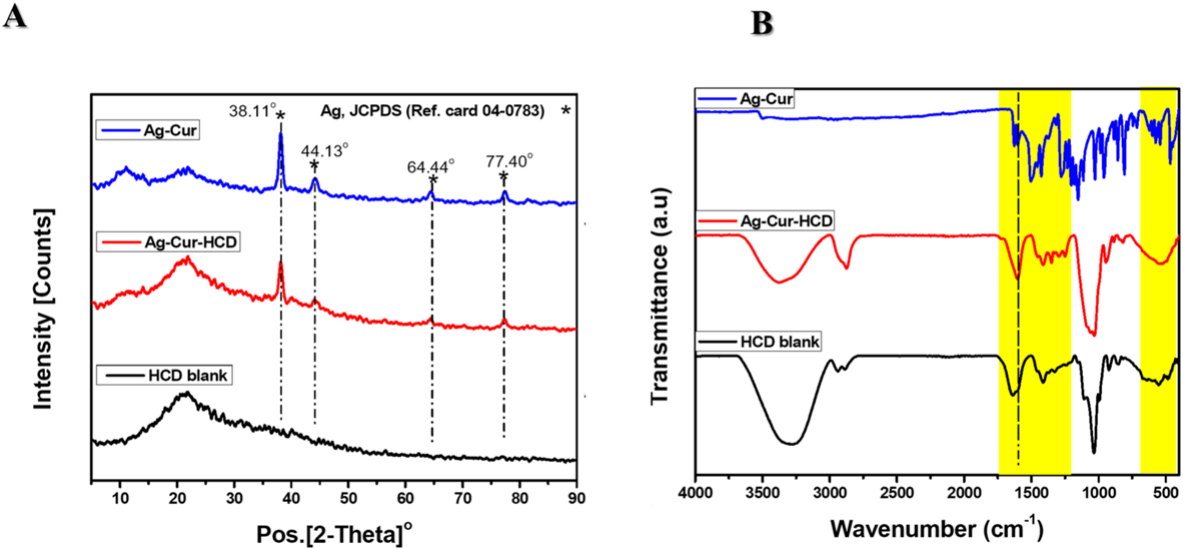
The (A) XRD pattern and (B) the FTIR spectra of HCD-blank, Ag-Cur, and Ag-Cur-HCD respectively.

The X-ray diffraction (XRD) analysis was employed to investigate the crystalline structure and phase composition of the prepared materials^56^. Figure 2A depicts the XRD patterns of the HCD blank, Ag–Cur, and Ag–Cur–HCD. The HCD blank displayed a broad diffraction peak centred around 2θ ≈ 22°, characteristic of the amorphous nature of the polymeric components (CMC, xanthan gum, and sodium alginate). The XRD pattern of the Ag–Cur–HCD composite further confirmed the successful embedding of Ag NPs within the polymer network. Distinct diffraction peaks appeared at 2θ = 38.11°, 44.13°, 64.44°, and 77.40°, assigned to the (111), (200), (220), and (311) planes of silver, respectively. These reflections are consistent with the face-centred cubic (fcc) structure of Ag NPs (JCPDS file No. 04-0783))^57,58^. The presence of these characteristic peaks confirms the formation of well-defined Ag NPs within the HCD matrix, confirming their successful incorporation into the polymeric hydrocolloid system. Compared to Ag-Cur XRD pattern, a noticeable reduction in peak intensity was observed upon incorporation of Ag NPs into HCD, suggesting possible interactions between Ag NPs and the host polymeric matrix. This interaction may enhance inter- and intramolecular attractions, resulting in a partial softening or structural reorganization of the polymer backbone. Furthermore, Fig. 2B. presents the FTIR spectra of the HCD blank, Ag-Cur, and Ag-Cur-HCD samples. For HCD blank, the broad peak at ~ 3281 cm⁻¹ corresponds to the stretching vibration of hydroxyl (–OH) groups, while the asymmetric stretching of CH₂ appears near 2939 cm⁻¹^59^. The carboxylate anion (–COO⁻) displayed two characteristic absorption bands at 1639 and 1412 cm⁻¹, assigned to asymmetric and symmetric stretching vibrations, respectively^60,61^. The band at 1034 cm⁻¹ attributed to C–O stretching in the acetyl groups of the sodium alginate backbone. For CMC, characteristic peaks were observed at 1603 cm⁻¹ and 1412 cm⁻¹, representing asymmetric and symmetric –COO vibrations^62^. The physical crosslinking between CMC and alginate reduce the intensity of the –OH groups peaks^63^. For Ag–Curcumin, a broad absorption at 3508 cm⁻¹ is associated with phenolic –OH stretching. Bands at 1620, 1427, and 1271 cm⁻¹ correspond to aromatic C–C–C, olefinic C–H, and phenolic C–O stretching, respectively, while a smaller peak at 1028 cm⁻¹ indicates asymmetric C–O–C stretching. The attenuation of these peaks suggests hydrogen bonding and surface interactions between curcumin and AgNPs^58^. Upon silver incorporation, most transmittance peaks decreased in intensity compared with the HCD blank, indicating reduced intermolecular hydrogen bonding and increased structural disorder within the polymeric chains. The broadening of the band near 550 cm⁻¹ reflects Ag incorporation. These findings align with XRD results, confirming a higher amorphous phase fraction in the Ag-modified CMC/CS matrix. Notably, the OH stretching peak shifted from 3281 to 3382 cm⁻¹, and the O–H bending peak from 1028 to 1031 cm⁻¹, further confirming silver–polymer interactions. Such shifts arise from alterations in potential energy distribution and intermolecular bonding within the matrix, highlighting the structural modification induced by Ag incorporation, these results were aligned with those obtained from other work^58^.

**Fig. 3:**
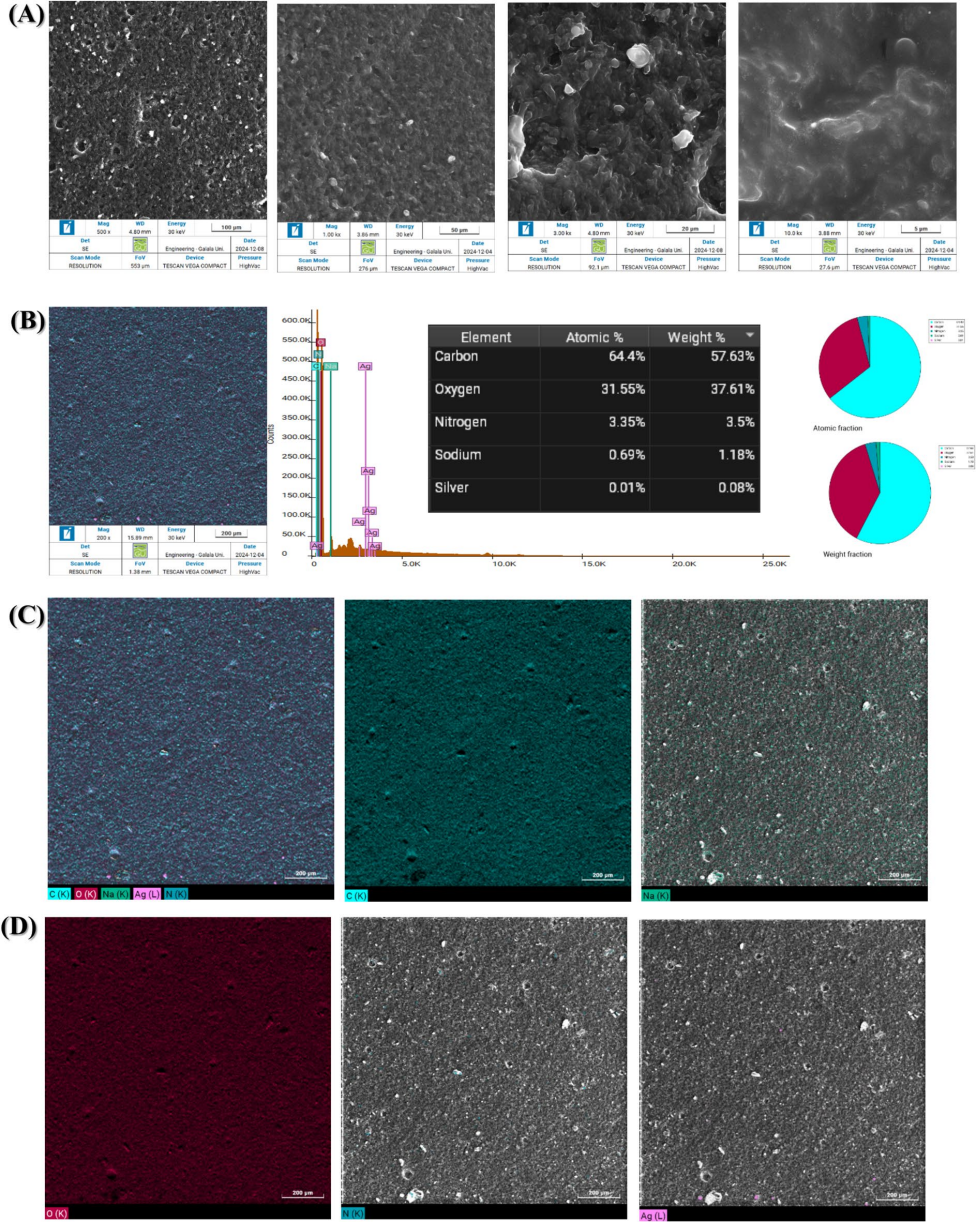
(A) The SEM images of Ag-Cur NPs hydrocolloid at different magnification, (B) the corresponding EDAX analyses, and C, D) The elemental Mapping images of Ag-Cur NPs hydrocolloid.

Additionally, the surface morphology and elemental composition of the Ag-Cur-HCD patch were examined via SEM-EDAX. SEM images at various magnifications (500×, 1000×, 3,000×, 5,000×, and 10,000×) were examined (Fig. 3A) and revealed a porous surface due to the presence of xanthan gum, which readily absorbs moisture and forms a porous structure. This porosity is advantageous for wound dressings, as it maintains an optimal moisture level at the wound site, creating a favourable environment for accelerated epithelial cell regeneration^64^. Additionally, EDAX analysis (Fig. 3(B)) confirms the presence of silver within the matrix. Furthermore, elemental mapping confirmed the homogeneous distribution of Ag-Cur within the polymer matrix (Fig. 3(C, D)). For the dressing to have consistent therapeutic activity, this is particularly important. The method’s simplicity of just incorporating and heating at 60 °C makes it scalable and cost-effective.

**Fig. 4:**
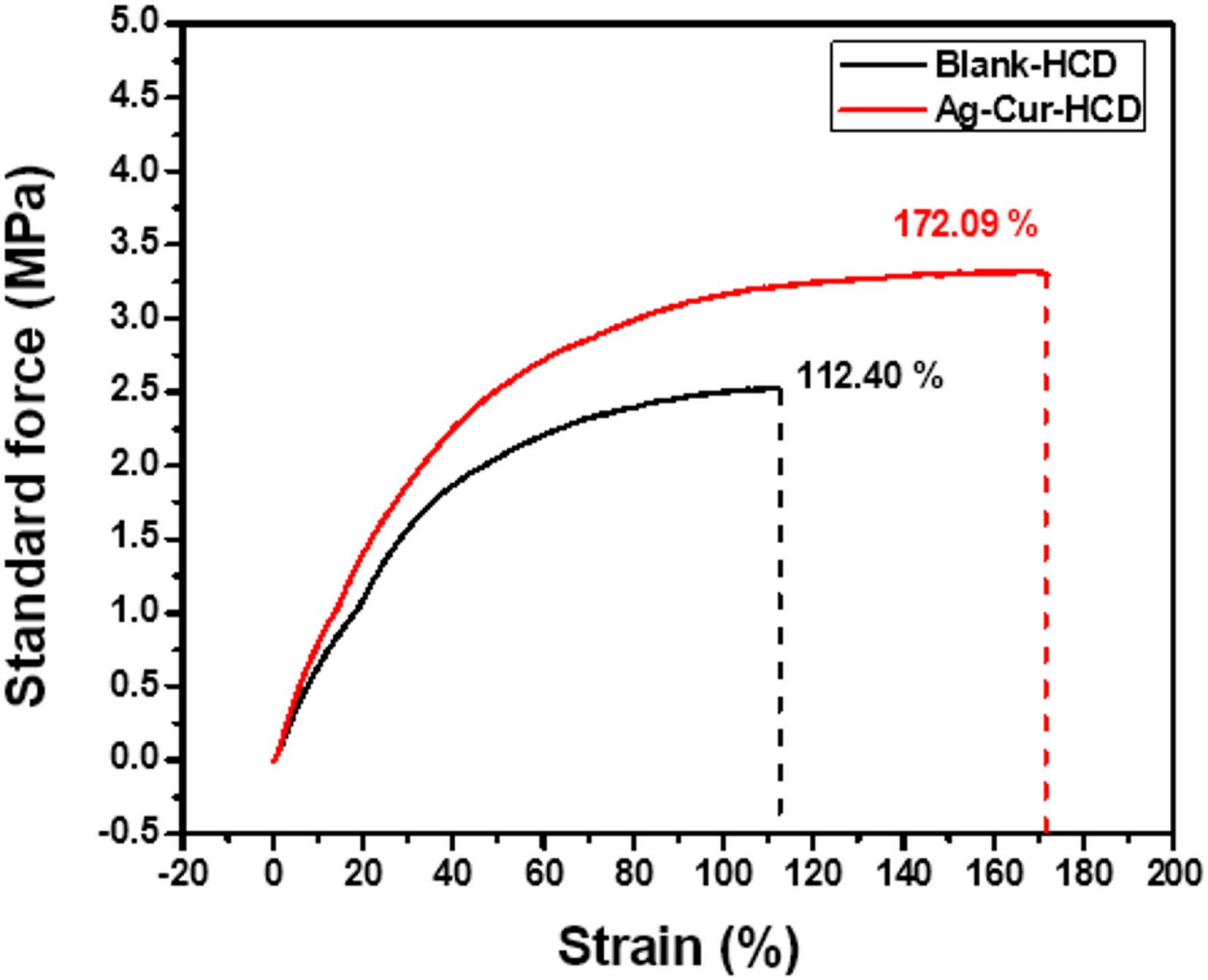
The mechanical properties stress-strain curve of loaded and unloaded Ag-Cur hydrocolloids. Data are presented as mean ± SD (*n* = 3 independent experiments).

The mechanical testing results demonstrated that (Fig. 4) the Ag-Cur-HCD exhibited markedly improved mechanical features compared to blank-HCD. Briefly, the Young’s modulus (E-modulus) increased from 4.76 MPa for blank-HCD to 6.43 MPa for Ag-Cur-HCD, confirming robustness and enhanced mechanical features after AgNP incorporation. Additionally, maximum tensile strength (Rm) enhanced from 2.52 MPa to 3.321 MPa and the stress break (RB) increased from 2.52 MPa to 3.30 MPa, confirming the load-bearing capacity of Ag-Cur-HCD. Furthermore, the elongation at break (e-break) improved from 112.40% to 172.09% and the strain at maximum (e-F max) improved from 111.49% to 166.58%, demonstrating a substantial improvement in flexibility and stretchability. Collectively, the obtained results indicate that improved integration between the polymeric matrix and Ag-Cur NPs not only reinforced the hydrocolloid matrix but also improved its deformability, producing an HCD with superior mechanical strength and elasticity. Furthermore, the Ag-Cur-HCD showed a higher mechanical strength in comparison to the DuoDERM™ (Young modulus < 1 MPa)^65^, indicating a strong network.

**Fig. 5:**
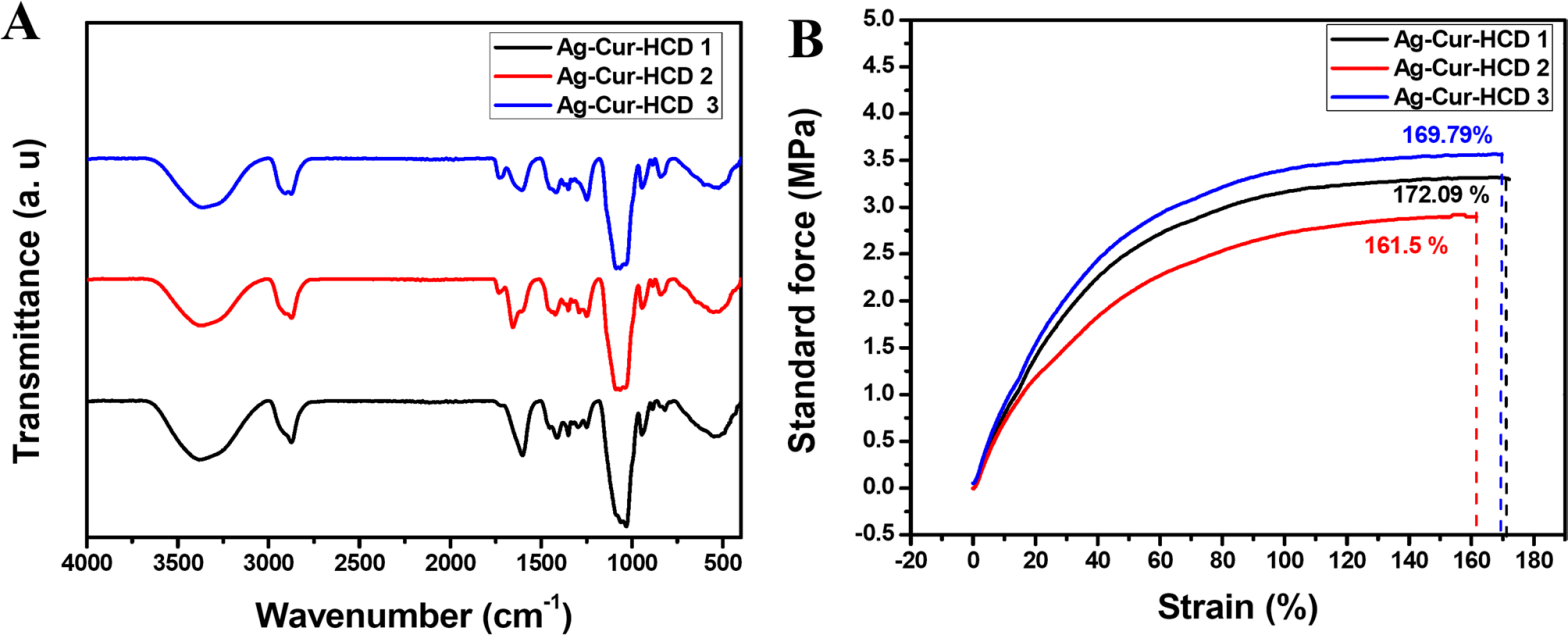
The FTIR spectra and The mechanical properties stress-strain curve of 3-different Ag-Cur NPs-HCD fabricated at the same conditions.

Additionally, to evaluate the batch-to-batch reproducibility of the developed Ag–Cur-HCD, three independently fabricated patches were characterized by FTIR spectroscopy and mechanical testing. The FTIR spectra of the three batches exhibited nearly identical characteristic absorption bands with no detectable peak shifts or the appearance of new bands, indicating a consistent chemical composition and preservation of the hydrocolloid network following the fabrication process (Fig. 5A). Furthermore, the mechanical properties showed only minor variation among the independently prepared patches, with elongation at break values of 172.09%, 169.79%, and 161.50%, demonstrating good repeatability of the manufacturing process (Fig. 5B). The small variation in mechanical performance is within the expected range for polymeric hydrogel systems and may be attributed to slight differences in polymer chain arrangement or water distribution during gel formation rather than changes in the chemical structure. Overall, the combined FTIR and mechanical results confirm the high reproducibility and manufacturing consistency of the Ag–Cur-HCD formulation, supporting the reliability of the preparation method for subsequent physicochemical and biological evaluations.

### Swelling capacity

**Fig. 6:**
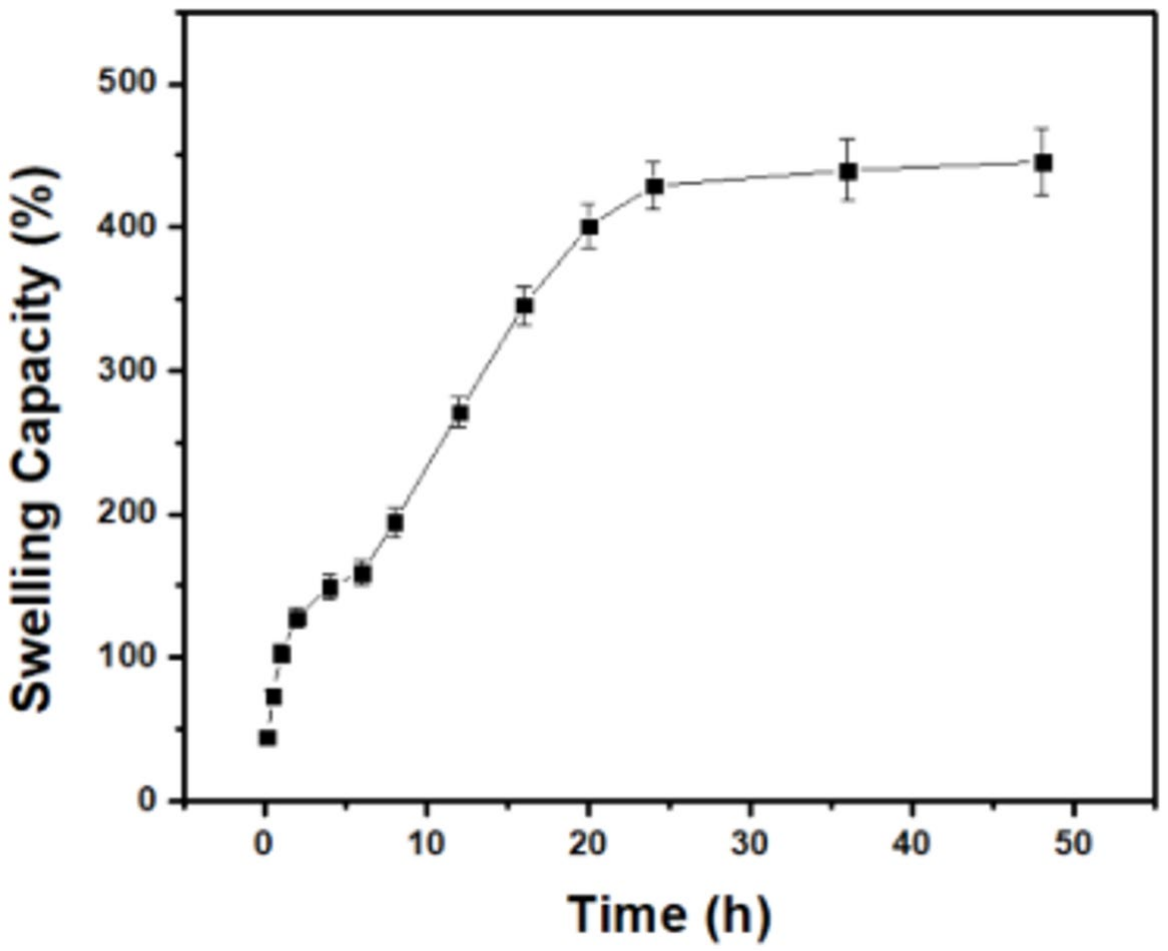
Swelling Capacity for Ag-Cur hydrocolloid. Data are presented as mean ± SD (*n* = 3 independent experiments).

The swelling capacity was conducted to assess the hydrocolloid’s ability to absorb wound exudate, a key requirement for managing chronic wounds with moderate to high exudate levels. The swelling capacity of the curcumin-silver nanoparticle-loaded hydrocolloid dressing (Ag-Cur- HCD) was significant during the first 1 h of the swelling test, increasing rapidly to approximately 103.07%. The swelling capacity then gradually increased from 103.07% to 428.76% over the next 24 h, stabilizing after ≈ 24 h of immersion in phosphate-buffered saline (PBS), and then the swelling ratio remains constant over 50 h (Fig. 6). The Ag-Cur-loaded HCD exhibited superior swelling capacity compared to a commercial hydrocolloid dressing (DuoDERM™, ConvaTec Inc.), achieving a swelling capacity of 428.76% versus 158.7% for DuoDERM™ or 340% for Acticoat^♦^ commercial products^65^. This enhanced swelling capacity is attributed to the incorporation of sodium alginate (SA), carboxymethyl cellulose (CMC), and xanthan gum, which are known for their high swelling capacities^66,67^. The Ag-Cur-loaded HCD exhibited a higher swelling capacity than a commercial hydrocolloid dressing (DuoDERM™, ConvaTec Inc.) (428.76% vs. 158.7%).

### In vitro release study

**Fig. 7:**
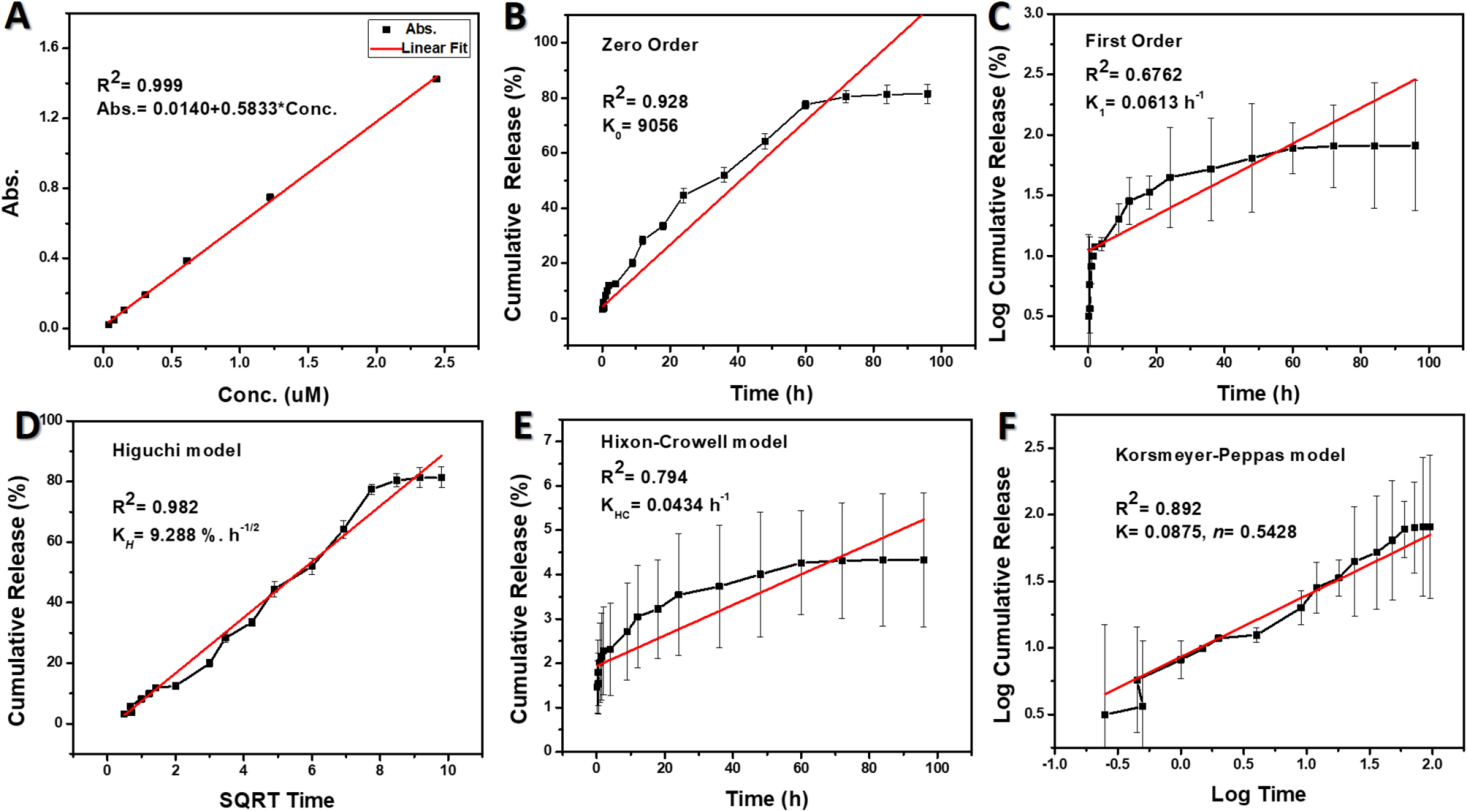
In vitro release kinetics of Ag–Cur nanoparticles from the hydrocolloid film at PBS 7.4 contains 1% tween 80. (A) Calibration curve of curcumin (R² = 0.999). (B–F) Kinetic models of cumulative release: (B) Zero-order, (C) First-order, (D) Higuchi, (E) Hixson–Crowell, and (F) Korsmeyer–Peppas models. Data are presented as mean ± SD (*n* = 3 independent experiments).

The in vitro release study was performed to evaluate the release kinetics of Ag-Cur nanoparticles from the hydrocolloid, using PBS with 1% Tween 80. The calibration curve for Ag-Cur NPs in PBS (pH 7.4) with 1% (w/w) Tween 80 was measured at the predetermined λ_max_ as shown in Fig. 7A, demonstrating a high linearity with an R² value of 0.999, indicating excellent accuracy in the dilution and measurement processes. The release profile of Ag-Cur NPs from the hydrocolloid was evaluated using the total immersion method over 100 h in PBS (pH 7.4) containing 1% (w/w) Tween 80, under continuous stirring (200 rpm) at 37 ± 2°C. The cumulative release curve showed a sustained release, with 30% of the Ag-Cur NPs released from the hydrocolloid within 20 h, reaching 80% after 100 h. Furthermore, the release kinetics of Ag–Cur nanoparticles from the hydrocolloid matrix were investigated using several mathematical models, including zero-order (Fig. 7B), first-order (Fig. 7C), Higuchi (Fig. 7D), Hixson–Crowell (Fig. 7E), and Korsmeyer–Peppas (Fig. 7F). Among these, the Higuchi model exhibited the best correlation with experimental data (R² = 0.982), indicating that the release is primarily induced by a diffusion-controlled process. The zero-order (R² = 0.928) and Korsmeyer–Peppas (R² = 0.892) models also showed good agreement, suggesting a near-constant release rate over time with minor contributions from polymer relaxation or swelling. In the Korsmeyer–Peppas model, the release exponent (*n*) was between 0.45 and 0.89 (0.5428), confirming a non-Fickian or anomalous transport mechanism. The hydrocolloid matrix gradually hydrates and swells upon immersion in the release media, increasing free volume and promoting diffusion of the integrated Ag-Cur NPs while maintaining the dressing’s structural integrity. The choice of the Higuchi model as the best explanation of the release behavior is further supported by the computed kinetic constants (k) and the matching R^2^ values for each of the models under investigation. Because it can maintain extended local therapeutic concentrations of the antimicrobial and anti-inflammatory agents, minimize burst release, lower the frequency of dressing replacement, and possibly lower the risk of local cytotoxicity associated with excessive drug exposure, diffusion-controlled sustained release is especially beneficial for wound-healing applications. Consequently, the release of Ag–Cur NPs from the hydrocolloid film can be characterized as a predominantly diffusion-controlled process with secondary swelling-controlled effects. These results are consistent with previous report^68^.

### Biological assays

The Ag-Cur hydrocolloid dressing’s therapeutic efficacy and safety were assessed using biological tests including anti-inflammatory, antimicrobial, and cytotoxicity.

#### Anti-inflammatory activity

**Fig. 8:**
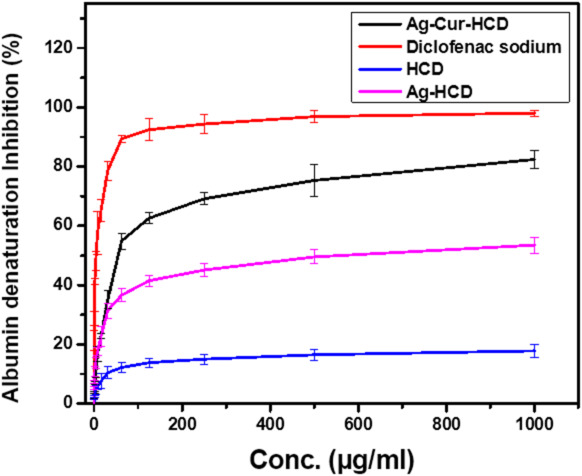
Albumin denaturation inhibition (%) for diclofenac, Ag-Cur hydrocolloid, and hydrocolloid blank. Data are presented as mean ± SD (*n* = 3 independent experiments).

The albumin denaturation inhibition assay was employed to evaluate the anti-inflammatory potential of the hydrocolloid, as protein denaturation is a key event in inflammation. Measurements were compared against various concentrations of diclofenac sodium and processed similarly to determine absorbance. As shown in Fig. 8, the curcumin-silver nanoparticle-loaded hydrocolloid (Ag-Cur hydrocolloid) exhibited the highest albumin denaturation inhibition of 82.43% ± 3.02% at a concentration of 1000 µg/mL, with an IC_50_ of 54.73 ± 0.95 µg/mL. While the blank hydrocolloid demonstrates 17.842% at the same concentration, indicating limited intrinsic anti-inflammatory capacity of the polymeric matrix itself, the diclofenac standard drug shows a 98.03% ± 1.05%. The Ag-Cur hydrocolloid consistently demonstrated superior inhibition across all tested concentrations. It displayed concentration-dependent inhibition of albumin denaturation, with increasing sample concentrations correlating with higher inhibition percentages. The Ag-Cur hydrocolloid showed a strong inhibitory effect at all concentrations, achieving a peak inhibition of 82.43% at 1000 µg/mL and significant activity even at lower concentrations (1.97% at 0.5 µg/mL).

**Fig. 9:**
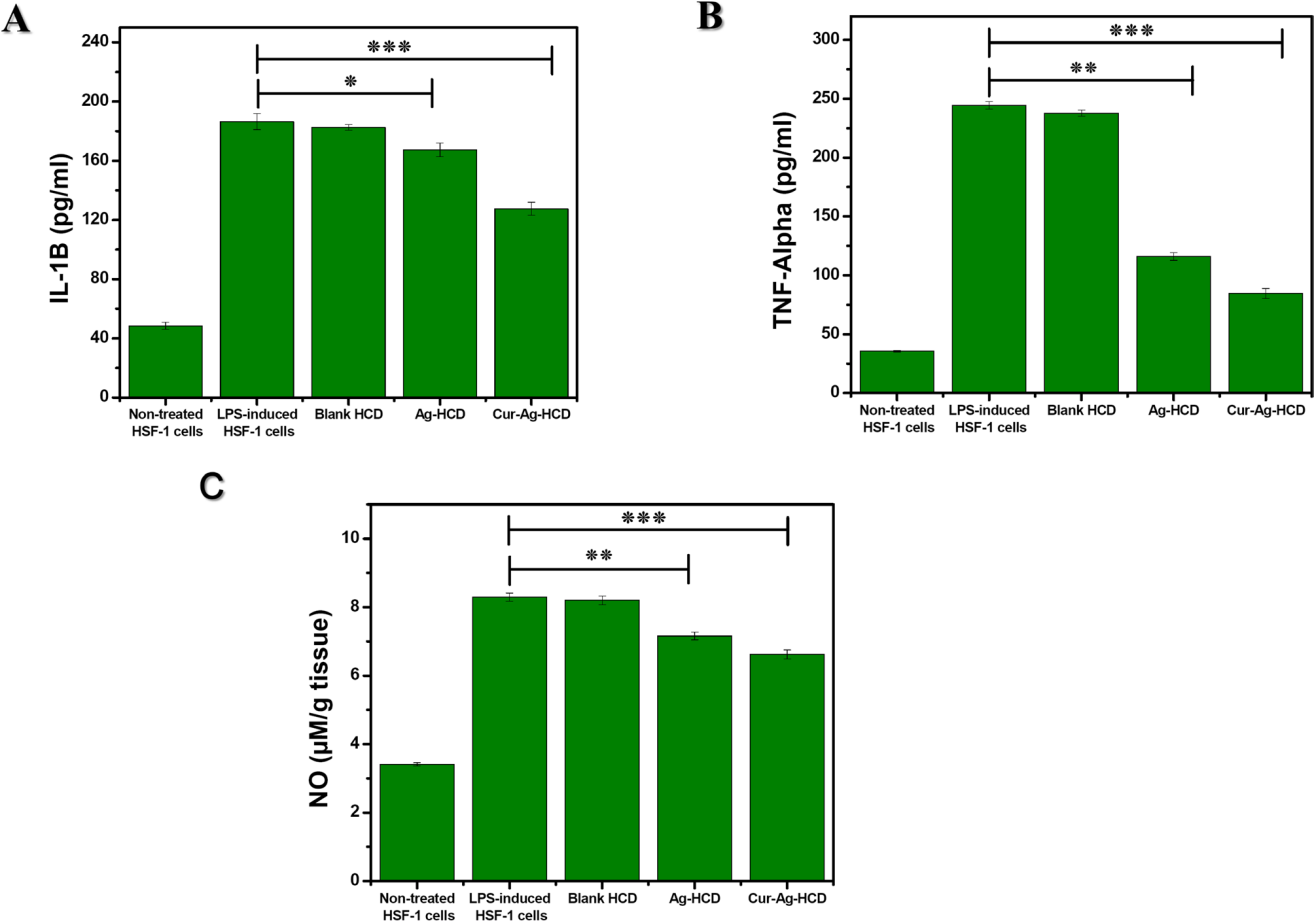
Effect of as-prepared HCDs on *TNF-Alpha* (A), interleukin-1β (*IL-1β*) (B), and Nitric oxide level (C) produced by LPS-stimulated macrophages. Data are presented as mean ± SD (*n* = 5 independent experiments). (*p < 0.05, **p < 0.01, and ***p < 0.001)

Additionally, as illustrated in Fig. 9, LPS-treated HSF-1 cells exhibited the highest nitric oxide level, *IL-1β*, and *TNF-α*, indicating an elevated basal production of oxidative stress and inflammatory responses in fibroblasts^53^. the Ag-Cur-HCD significantly outperforms both untreated LPS control, HCD blank, and Ag-HCD in the *IL-1β* (Fig. 9A) dose-response-like reduction of LPS-induced inflammation in HSF-1 cells (*p < 0.05, **p < 0.01, and ***p < 0.001). The ~ 65% decrease (~ 244 to ~ 284 pg/mL, ***p < 0.001) with Ag-Cur-HCD was observed. The LPS treatment activates pro-inflammatory signalling pathways that are usually linked to LPS stimulation, leading to a high level of *TNF-α*. As shown in Fig. 9B, the blank HCD formulation didn’t display a significant reduction in *TNF-α*, indicating, that the HCD carrier alone didn’t show an anti-inflammatory effect. Conversely, cells subjected to Ag-HCD (**p < 0.01) and Ag-Cur-HCD (***p < 0.001) formulations exhibited a significant reduction in *TNF-α* level by reducing the level from 237.81 ± 2.5 to 84.55 ± 4.23 pg/mL, thereby illustrating their marked anti-inflammatory properties. *TNF-α* levels were most affected by Ag-Cur-HCD, which brought the cytokine concentration down to levels that were very similar to those in the untreated control group. The significant anti-inflammatory activity is likely driven by the synergistic effect of curcumin and silver, where curcumin suppresses inflammation through inhibition of NF-κB signalling and promotion of macrophage apoptosis^69^ while silver nanoparticles could reduce inflammation during RA progression by inhibiting pro-inflammatory cytokines such as IL-6 and CRP^70^. Furthermore, the results shown in Fig. 9C display a significant variance in nitric oxide (NO) levels among HSF-1 cells exposed to various treatments. NO level was lower in Ag-HCD-treated cells (7.16 ± 0.11 µmol/L, **p < 0.01) than in LPS-treated HSF-1 cells, suggesting a modest NO-suppressive effect that is probably due to AgNPs’ well-known antioxidant action^71^. While Ag-Cur-HCD treatment significantly reduced nitric oxide level (6.62 ± 0.13 µmol/L, ***p < 0.001) and produced the lowest NO level of all the groups examined.

Based on these results, the combined effects of silver nanoparticles and curcumin may be responsible for the improved ability to downregulate inflammatory and oxidative biomarkers in Ag-Cur-HCD. Silver nanoparticles provide further antibacterial and anti-inflammatory properties, and curcumin is known to disrupt inflammatory signaling pathways like NF-κB as illustrated in previous works^70,72^. Overall, these results highlighting Ag-Cur-HCD’s potential as a promising wound dressing patch for controlling inflammation and promoting improved cellular recovery.

#### Antimicrobial activity

As illustrated in Table 1, while the HCD blank had no effect on any of the tested microorganisms, the Ag-Cur-HCD exhibited significant antibacterial properties against the fungus *Candida albicans*, Gram-negative bacteria * (Escherichia coli and Pseudomonas aeruginosa)*, and Gram-positive bacteria *(Staphylococcus epidermidis and Staphylococcus aureus).* The results were consistent with earlier research^73,74^. Briefly, Gram-positive bacteria were more susceptible to the Ag-Cur hydrocolloid than Gram-negative bacteria. As demonstrated previously, curcumin is more effective against Gram-positive bacteria than Gram-negative bacteria^75^. The antibacterial activity may be change based on the cell wall structure of bacteria; for example, Gram-negative bacteria possess an outer membrane surrounding a thin peptidoglycan layer that contains proteins, phospholipids, and lipopolysaccharides, Gram-positive bacteria have a thicker peptidoglycan layer^75,76^. The unique outer membrane structure of Gram-negative bacteria may hinder the penetration of certain antimicrobial agents. Hence, attributed to the size confinement of silver nanoparticles and their ability to penetrate bacterial cell walls, it could enhance the antimicrobial activity. Additionally, curcumin also possesses a bactericidal activity by generating ROS such as superoxide and hydrogen peroxide, which damage bacterial cell wall structures and cause cell death. Curcumin, an amphipathic and lipophilic compound, has been demonstrated in previous studies to bind to the bacterial cell wall and increase membrane permeabilization in *S. aureus* and *E. coli*, resulting in cell membrane damage and lysis^77,78^.

**Fig. 10:**
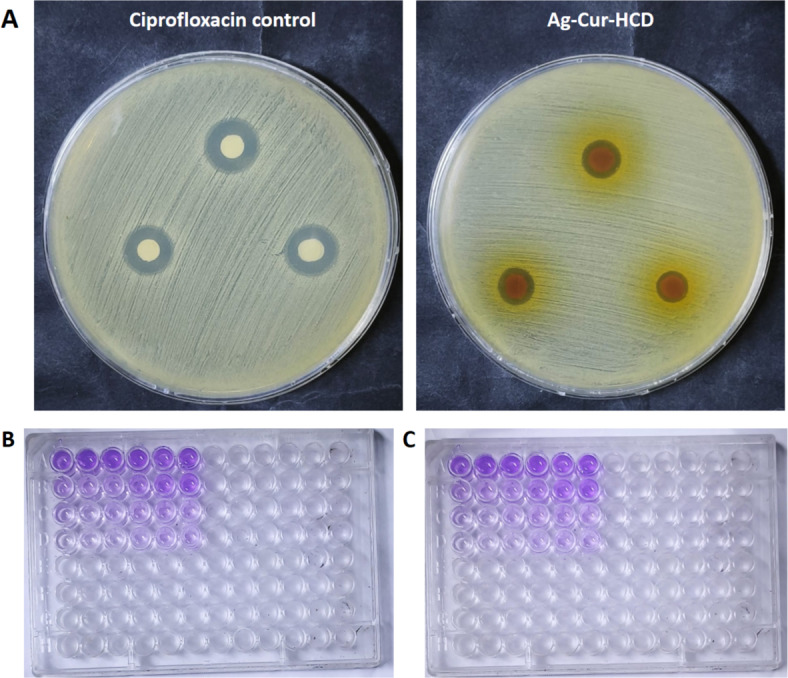
Antibacterial evaluation against methicillin-resistant Staphylococcus aureus (MRSA). (A) Agar well diffusion assay showing the inhibition zones produced by ciprofloxacin (control) and Ag–Cur-HCD. (B) Broth microdilution assay of ciprofloxacin control for determination of MIC/MBC. (C) Broth microdilution assay of Ag–Cur-HCD for determination of MIC/MBC against MRSA. Data are presented as mean ± SD (*n* = 3 independent experiments).

Agar diffusion, MIC/MBC, and anti-biofilm tests verified the new Ag–Cur-HCD formulation’s significant antibacterial efficacy against MRSA. Ag–Cur-HCD exhibited a mean inhibition zone of 9.60 ± 0.216 mm in the agar diffusion test, demonstrating the formulation’s capacity to stop MRSA growth (Fig. 10). This inhibitory zone supports the developed hydrocolloid system’s antibacterial capability even if it was smaller than the control’s (13.13 ± 0.205 mm). According to the broth microdilution data, Ag–Cur-HCD demonstrated bactericidal action at an MBC of 7.8 µg/mL and inhibited MRSA growth at a MIC of 15.62 µg/mL, resulting in an MBC/MIC index of 2 (Table 2). Additionally, Ag–Cur-HCD demonstrated significant anti-biofilm activity in addition to its direct antibacterial action, with inhibition percentages of 92.03%, 82.45%, and 54.22% at 75%, 50%, and 25% of MBC, respectively (Table 3). The formulation may interfere not only with planktonic bacterial growth but also with bacterial adhesion and early biofilm establishment, which are crucial stages in chronic wound colonization, according to the significant suppression of MRSA biofilm formation at sub-MBC concentrations. Because MRSA biofilms function as protective microbial communities that withstand antimicrobial treatment and extend the inflammatory phase of wound healing, this phenomenon highly significant.

Mechanistically, the synergistic action of curcumin and silver in the hydrocolloid dressing may be responsible for the antibacterial and anti-biofilm properties. While curcumin has been shown to have antibacterial, anti-inflammatory, and biofilm-modulating properties, silver is known to damage bacterial membranes, change protein function, and cause oxidative stress. When taken as a whole, these activities might reduce bacterial colonization, prevent the growth of biofilms, and reduce inflammation driven by infections at the wound site. Ag–Cur-HCD may therefore offer a more appropriate local environment for fibroblast proliferation, extracellular matrix deposition, and re-epithelialization, facilitating the process of wound healing.

Overall, Ag–Cur-HCD’s dual bactericidal and anti-biofilm efficacy against MRSA demonstrates its potential as a multipurpose wound dressing that may address bacterial infection and biofilm persistence, two critical barriers to chronic wound healing.

**Table 1:** The antimicrobial activity and Mean zone of inhibition in mm produced on a range of pathogenic microorganisms of the Ag-Cur hydrocolloid, and controls against different micro-organisms.

Tested microorganisms	Sample code		
	HCD	Ag-Cur-HCD	Control
FUNGI			Ketoconazole
*Candida albicans* RCMB 005003 (1) ATCC 10231	NA	18 ± 1.21	20 ± 1.0
Gram positive bacteria:			*Gentamycin*
*Staphylococcus aureus* ATCC 25923	NA	28 ± 2.32	24 ± 1.55
*Staphylococcus epidermidis* RCMB 009 (2)	NA	36 ± 3.10	28 ± 1.80
Gram negative bacteria:			*Gentamycin*
*Escherichia coli* ATCC 25922	NA	32 ± 2.95	30 ± 2.10
*Pseudomonas aeruginosa* ATCC 27853	NA	26 ± 2.10	27 ± 2.0

*NA: NO activity. Data are presented as mean ± SD (*n* = 3 independent experiments).

**Table 2:** The antibacterial activity against methicillin-resistant Staphylococcus aureus (MRSA) was evaluated using broth microdilution and agar well diffusion assays.

Tested sample	Inhibition zone (mm) (Three Readings)			Mean inhibition zone diameter (mm)	MIC (µg/mL)	MBC (µg/mL)
	1st reading	2nd reading	3rd reading			
Ag-Cur-HCD	9.3	9.7	9.8	9.6 ± 0.216	7.8	15.62
Control	13.4	12.9	13.1	13.13 ± 0.205	31.25	62.5

The results were expressed as minimum bactericidal concentration (MBC), minimum inhibitory concentration (MIC), and inhibition zone diameter (mm). Values for the inhibition zone are expressed as mean ± SD (*n* = 3).

**Table 3:** Anti-biofilm activity against methicillin-resistant Staphylococcus aureus (MRSA) at sub-bactericidal concentrations is measured as the percentage of biofilm inhibition (%). The data are displayed as mean ± SD (*n* = 3).

Anti-Biofilm for MRSA	Control		Ag-Cur-HCD	
	Anti- biofilm activity %	S.D. (±)	Anti- biofilm activity %	S.D. (±)
Blank (Media only)		0.001		0.001
Media + Organism(Cont.)	-	0.049	-	0.049
75% of MBC	96.76	0.006	92.03	0.008
50% of MBC	86.93	0.006	82.45	0.010
25% of MBC	75.32	0.018	54.22	0.009

#### Cytotoxicity assay

**Fig. 11:**
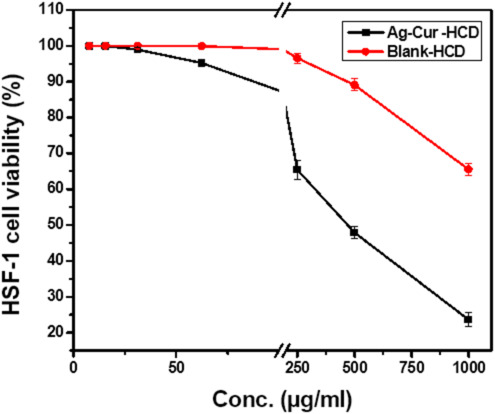
Dose-dependent toxicity profile of Blank-HCD and Ag-Cur-HCD against HSF-1cell line by MTT assay.

The in vitro cytotoxicity of the fabricated hydrocolloids was presented in Fig. 11. Human skin fibroblasts (HSF-1) were used to assess the HCD matrix’s cytotoxicity; however, no cytotoxic effects were found. The result of blank-HCD (CC_50_> 100%) is consistent with earlier research demonstrating the biocompatibility nature of sodium alginate (SA)-based films, demonstrating no effect on the viability of L929 mouse fibroblasts or human fetal foreskin fibroblasts^79^. Similarly, Das et al. (2023) demonstrated that CMC–SA composite films reinforced with nano silver (Ag) and graphene oxide exhibited no cytotoxicity toward non-tumorigenic human mammary epithelial cells (MCF-10 A)^80^. These consistent findings highlight the inherent biocompatibility of polysaccharide-based hydrocolloid matrices, confirming their safety and use as an effective wound dressing matrix. Furthermore, the Ag-Cur - HCD was showed minimal cytotoxicity against normal healthy HSF-1 cells. Additionally, the evaluated Ag-Cur-HCD’s cytotoxicity profile against HSF-1 cells showed a concentration-dependent cytotoxic effect, suggesting that the Ag-Cur-HCD had more cytotoxic effects at higher concentrations. Ag-Cur-HCD maintained remarkably high cell viability at lower doses, but as the concentration increased, a progressive drop was seen, reaching about 23% viability at 1000 µg/mL. At lower doses, the calculated CC_50_ value of 47.06 ± 2.309% shows a very mild level of cytotoxicity and moderate cytocompatibility. To maintain therapeutic efficacy and assure cellular safety, the formulation should ideally be employed at doses much below the CC_50_ value. For topical medicinal solutions to be developed safely and effectively, biological activity and minimal cytotoxicity must be balanced. These results confirm that the Ag-Cur -HCD is safe below 470.06 ± 23.09 µg/mL and suitable for further evaluation in in vivo studies. While other studies have assessed cyto-compatibility using cell lines like L929 fibroblasts or HaCaT keratinocytes^81,82^, the focus on HSF-1 cells is particularly relevant for chronic wound applications, as fibroblasts play a critical role in tissue repair and extracellular matrix formation^83^.

## Conclusion

In summary, we successfully prepared bioactive curcumin-silver nanoparticles (Ag-Cur NPs) and incorporated them into a hydrocolloid membrane through a simple casting-evaporation technique composed of alginate, carboxymethyl cellulose (CMC), and xanthan gum. This integration enhanced the biological activities associated with wound healing, including antimicrobial and anti-inflammatory, hence promoting the healing process. Physicochemical, mechanical, and morphological characterization demonstrated that the hydrocolloid membrane is suitable for use as a wound dressing due to its adequate mechanical strength, capacity to absorb wound exudate, and ability to maintain a moist wound environment. The developed Ag–Cur NPs maintained acceptable physicochemical stability after three months of storage under ambient conditions indicating a long-term stability and the fabricated Ag-Cur-HCD demonstrated good batch-to-batch reproducibility, supporting the robustness and reliability of the developed wound dressing. The sustained release of Ag-Cur NPs enhanced the membrane’s anti-inflammatory properties. Ag–Cur-HCD demonstrated significant antibacterial, bactericidal, and anti-biofilm activity against MRSA, highlighting its potential as a multifunctional wound dressing for infection management and reducing biofilm-related healing delay. moderate cytocompatibility with HSF-1 cells, allowing it to be used at concentrations below the CC_50_ limit. Additionally, Ag-Cur-HCD succeeds over both Blank-HCD and Ag-HCD in suppressing LPS-induced inflammation and oxidative stress in HSF-1 cells, as seen by the *IL-1β*,* TNF-Alpha*, and NO levels. As a result, Ag-Cur-HCD considerably modulates important inflammatory and antioxidant pathways. Thus, the hydrocolloid membrane developed in this study exhibits promising results and has the potential to serve as an effective bioactive wound dressing. The future work will focus on evaluating the developed HCD patches using in vivo model of infected and chronic wounds, as well as long-term safety and comparative efficacy assessments.

## Data Availability

All data generated or analyzed during this study are included in this published article, which are available from the corresponding author on reasonable request.
